# Implementing Electronic Health Records in Philippine Primary Care Settings: Mixed-Methods Pilot Study

**DOI:** 10.2196/63036

**Published:** 2025-07-15

**Authors:** Anton Elepaño, Carol Stephanie Tan-Lim, Mark Anthony Javelosa, Regine Ynez De Mesa, Mia Rey, Josephine Sanchez, Leonila Dans, Antonio Miguel Dans

**Affiliations:** 1 Nuffield Department of Primary Care Health Sciences, Medical Sciences Division, University of Oxford, Radcliffe Observatory Quarter, 43 Woodstock Road, Oxford, OX2 6GG, United Kingdom, 44 7879663695; 2 Program on Health Systems Development, Center for Integrative and Development Studies, University of the Philippines Diliman, Quezon City, Philippines; 3 Department of Clinical Epidemiology, College of Medicine, University of the Philippines Manila, Manila, Philippines; 4 Department of Accounting and Finance, Cesar E.A. Virata School of Business, University of the Philippines Diliman, Quezon City, Philippines

**Keywords:** electronic health records, medical informatics, digital health, implementation report, electronic health records system

## Abstract

**Background:**

Between 2020 and 2022, the Philippine Primary Care Studies program, a government-funded initiative supporting universal health care implementation, piloted two electronic health records (EHR) systems across urban, rural, and remote primary care sites.

**Objective:**

The study aimed to evaluate the implementation of two EHR systems in diverse primary care settings in the Philippines over a three-year period.

**Methods:**

This implementation study used an explanatory mixed methods design. Two EHR systems were deployed: an Open Medical Records System (OpenMRS)-based platform in 2016, and a Microsoft-based system in 2021. Both systems integrated clinical documentation, pharmacy, laboratory, and reporting modules. Implementation strategies included training workshops and materials, iterative user feedback loops, and infrastructure cofinancing with local governments. Surveys were administered yearly to all end users. The primary outcome was behavioral intention to use the system. Quantitative data were supplemented by inductive content analysis of qualitative responses to explain observed trends.

**Results:**

A total of 351 survey responses were collected from 2020 to 2022. In 2020, the intention to use the OpenMRS-based EHR was high across all sites. By 2022, following the launch of the Microsoft-based EHR, acceptability declined significantly among doctors and administrative staff, particularly at the urban site. In contrast, the remote site which retained the OpenMRS-based system maintained high acceptability levels. Qualitative findings revealed that while the new EHR system provided a more privacy-focused design, users preferred a cross-platform EHR to allow more flexible access to patient data. At the rural site where the EHR was used to facilitate task-shifting among nurses involved in clinical management, users were less impacted by this shift.

**Conclusions:**

The disparities in EHR acceptability across urban, rural, and remote sites were influenced by contextual, technical, and demographic factors. The decline in acceptability following the EHR system transition highlights the importance of implementation strategies that reflect the specific needs and capacities of each setting. These findings offer practical insights for adapting EHR systems to diverse primary care contexts.

## Introduction

Following the enactment of the Philippine Universal Health Care Law, which mandated the national implementation of electronic health records (EHR) for primary care providers [1], the adoption of EHR systems has faced multifaceted challenges encompassing sociotechnical, organizational, and environmental barriers [2,3].

EHRs are considered digital health interventions intended to address multiple health system challenges [4]. These challenges include poor data quality, underutilization of health care, and operational inefficiencies related to paper-based clinical documentation and insurance claims processing [5,6]. EHRs also aim to address transportation and communication barriers, human resource shortages, and referral inefficiencies, particularly by facilitating task-shifting models in which nonphysician providers such as nurses and midwives assume expanded clinical roles in remote areas [7].

Despite the development of many EHR systems tailored for rural primary care facilities in the Philippines, adoption remains limited [2] and implementation reports or user-centered evaluations are scarce [8]. However, when successfully implemented, EHRs can enhance the collection, storage, and retrieval of health information across clinical, paramedical, and administrative domains [4].

To provide recommendations for the widespread implementation of EHR systems, the Philippine Primary Care Studies (PPCS) initiated a digitalization program in three pilot sites that reflect the country’s primary care contexts: urban (Quezon City, National Capital Region), rural (Samal, Bataan), and remote (Bulusan, Sorsogon). This initiative aligned with the national digital health strategy, which designated EHR adoption as a foundational step in building an integrated health information system to support evidence-based policy and health financing decisions [1].

Initial surveys during the first year of program implementation revealed high EHR acceptability across a diverse range of users and settings [5]. Since then, both the contextual landscape and system features have evolved. Some rural facilities have reverted to or continued using paper-based systems [8]. This study provided a comprehensive reassessment of how various factors have shaped the EHR implementation over time.

## Methods

### Study outcomes

The overall aim of the implementation was to assess how EHR systems could be effectively used across various primary care contexts. The primary outcome was user acceptability, operationalized as continued intention to use the system [9]. Distal outcomes such as sustainability and clinical outcomes were beyond the scope of this study. The secondary objective was to explain variations in acceptability by delving into contextual factors influencing system adoption. This study adhered to the iCHECK-DH (Checklist for the Reporting on Digital Health Implementations) guidelines [10] and the GRAMMS (Good Reporting of A Mixed Methods Study) reporting standards [11].

### Blueprint Summary

The digital health interventions integrated several components including electronic medical records, health management information systems, laboratory and diagnostics information, and pharmacy information [4]. Two EHRs were implemented across PPCS pilot sites, one in 2016 and another in 2021. Training was delivered through workshops and asynchronous instructional materials. Broader implementation strategies included internet infrastructure deployment (in collaboration with the local government units), and continuous feedback loops between system developers and users. Prescribing and laboratory data were integrated into the EHR to monitor costs and support the development of a primary care benefits package [12].

### Technical Design

The first EHR system, built on the open-source Open Medical Records System (OpenMRS) platform was introduced in 2016 at an urban site, and subsequently in 2019 at the rural and remote sites. Developed in-house by the PPCS team, this system connected primary care services, laboratory units, and pharmacies. Main features included triage and queueing modules, clinical notes accessible only to authorized providers, laboratory request and result interfaces, pharmacy dispensing modules, and administrative reporting tools.

The second EHR system, launched in April 2021 for the urban and rural sites, used a Microsoft-based desktop and cloud infrastructure. This Microsoft-based EHR was not implemented at the remote site due to the lack of high-speed broadband internet. Developed by a government-owned digital solutions subsidiary, this system introduced role-based access for nonphysician providers to draft clinical notes and propose management plans for physician approval. Additional functionalities included real-time referral notifications and a redesigned user interface. Core features remained consistent with the OpenMRS-based EHR platform.

### Target

The three primary care settings of the PPCS program were selected to represent urban, rural, and remote contexts. Rural sites are defined by the National Statistics Office as municipalities with a population density of fewer than 1000 people per square kilometer [13], while remote sites are designated by the Department of Health as geographically isolated and disadvantaged areas [14]. The urban site, located within a public university in a high-income municipality in Quezon City, provided primary care services to approximately 15,051 faculty members, employees, and their dependents. The rural site, situated in a low-income municipality in Bataan, served 35,298 residents with a population density of 627 inhabitants/km^2^ [15,16]. The remote site, located in a low-income geographically isolated mountainous area in Sorsogon, catered to 22,884 residents (238 inhabitants/km^2^) [16,17].

End users included administrative personnel, community health workers, nurses, midwives, physicians, dentists, nutritionists, pharmacists, and laboratory technicians. These users were responsible for data entry, service provision, and generating reports for local and national reporting.

### Data

Patient data were entered through password-protected accounts and stored on secure servers managed by the system providers. Data sharing was restricted to health professionals managing the same patients. Data privacy protocols conformed to the Data Privacy Act of 2012, which grants patients the right to be informed, access, and modify their data [18]. Third-party use of data for research required clearance under the National Ethics Guidelines for Health and Health-Related Research (2017) [19].

### Interoperability

Both systems adhered to national data standards issued by the Department of Health [1]. The OpenMRS-based EHR supported Fast Healthcare Interoperability Resources, while the Microsoft-based version did not. Clinical notes were primarily composed of sectioned free-text fields (eg, chief complaint, history of present illness). Diagnoses were entered using *ICD-10* codes via auto-populated dropdown menus. Similarly, prescriptions were entered using dropdown lists based on the national drug formulary.

### Participating Entities

The program was implemented by PPCS under the Program on Health Systems Development, University of the Philippines Center of Integrative and Development Studies. Funding was provided by the Department of Health, the Philippine Health Insurance Corporation, the Emerging Interdisciplinary Research Program, and the Philippine Council on Health Research and Development. The Microsoft-based EHR was developed through the Technology Transfer and Business Development Office of the University of the Philippines Diliman. This EHR was subsequently co-owned by the system provider and the university.

### Budget Planning

The total cost of the project was estimated at Php 6,000,000 (USD 104,572). Capital costs were cofinanced by local government units and the implementing agency. This covered internet infrastructure (ten internet towers amounting to Php 800,000 (USD 13,943)), network equipment (routers and cables worth Php 40,000 (USD 697)), EHR development (developers’ salaries over Php 1,000,000 (USD 17,429)), and workstations (desktops, laptops, and printers worth Php 2,000,000 (USD 34,857)). Recurring costs for IT support personnel were approximately Php 800,000 (USD 13,943) annually. Licensing costs were not incurred as both systems were developed in-house.

### Sustainability

Postpilot funding for both systems varied, depending on the discretion of local government units. Although this evaluation focused on acceptability rather than sustainability, the latter was implicitly linked to user endorsement. EHR users, who also served on local health boards, played advocacy roles in securing funding.

### Evaluation

A sequential mixed-methods design was used to assess user acceptability. Surveys were conducted annually from 2020 to 2022. An interpretivist approach underpinned the analysis, acknowledging the co-construction of meaning between researchers and participants. Survey design was guided by the Unified Theory of Acceptance and Use of Technology (UTAUT) [20].

Mixed methods allowed the team to capture the evolving nature of the intervention [21]. Quantitative data identified patterns in acceptability, whereas qualitative data provided context to these observations. Although data collection was concurrent, analysis occurred sequentially, with priority given to quantitative data. This sequential approach facilitated identification of relevant questions to guide the content analysis of qualitative data. Integrating datasets enabled corroboration and elaboration of findings.

All EHR users were invited to participate and provided written informed consent. The survey instruments featured a combination of Likert-scale questions and free-text fields. Survey validation and Unified Theory of Acceptance and Use of Technology model testing are detailed elsewhere [5]. Free-text responses were analyzed using semantic, inductive content analysis. Due to brevity of responses, coding was kept descriptive to highlight user experiences [22]. The final synthesis was reviewed by the core research team and the PPCS steering committee.

### Ethical Considerations

This study received ethics approval from the University of the Philippines Manila Research Ethics Board (2015-489-0) and the Department of Health Single Joint Research Ethics Board (2029‐55). Written informed consent was obtained from all participants. They were free to decline participation or withdraw from the study at any time. No financial or material compensation was provided for completing the questionnaire. Identifying information was removed during analysis to ensure participant anonymity.

## Results

### Coverage

Surveys were carried out at sites where the EHRs were implemented, covering 21 barangay health stations (14 in Samal and seven in Bulusan), two rural health units (one in Samal and one in Bulusan), and one university-affiliated clinic in Quezon City. The surveys achieved response rates of 53% (135/255) in 2020, 48% (127/263) in 2021, and 68% (89/131) in 2022. Respondents were predominantly female and were within the 31-60 age group (Table 1). Nurses and midwives accounted for the majority of respondents across all three sites, comprising 175 (50%) of the total sample. A total of 165 respondents (47%) reported having less than five years of service, while 221 (63%) reported more than 48 weeks of experience using the EHRs.

**Table 1.** Demographic characteristics of survey respondents.

Characteristics	Year 2020 (n=135), n (%)	Year 2021 (n=127), n (%)	Year 2022 (n=89), n (%)
Location			
Remote	45 (33)	43 (34)	24 (27)
Rural	48 (36)	39 (31)	42 (47)
Urban	42 (31)	45 (35)	23 (26)
Age (years)			
≤30	22 (16)	26 (21)	16 (18)
31‐60	101 (75)	93 (73)	66 (74)
>60	12 (9)	8 (6)	7 (8)
Sex			
Female	114 (84)	108 (85)	73 (82)
Male	21 (16)	19 (15)	16 (18)
Role			
Administrative staff	24 (18)	17 (13)	8 (9)
Doctor	16 (12)	16 (13)	24 (27)
Midwife/nurse	70 (52)	63 (50)	42 (47)
Paramedical staff	25 (19)	31 (24)	15 (17)
EHR^a^ use (months)			
≤12	76 (56)	20 (16)	40 (45)
13‐24	59 (44)	27 (22)	14 (16)
25‐36	0 (0)	1 (1)	5 (6)
>36	0 (0)	77 (62)	30 (34)

a EHR: electronic health record.

### Acceptability Surveys

**Figure 1.**
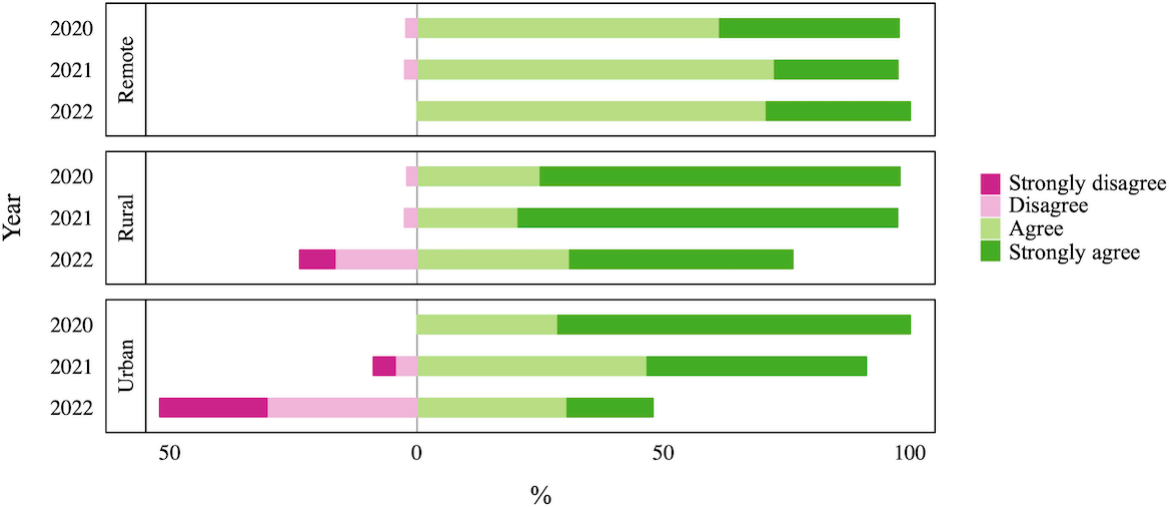
Percentage responses to the item “I intend to use the EHR in the next 12 months” revealing shifts in behavioral intention from 2020 to 2022 across pilot sites.

The acceptability of the EHR system showed distinct patterns over the three-year study period (Figure 1). In 2020, respondents generally expressed positive intention to use the OpenMRS-based EHR across the three sites. However, by 2021, with the transition to the new EHR system in the urban and rural sites, a noticeable shift in acceptability occurred, especially in the urban site, where some users expressed disagreement with the continued use of the EHR. By 2022, more than half of the urban site respondents disagreed with the continued use of the EHR. This trend was mirrored, though to a lesser extent, among rural users. The remote site users, who continued using the OpenMRS-based EHR, maintained consistent acceptability levels towards the EHR throughout the three years.

Of the 64 respondents (72%) in the 2022 endline survey who also participated in the 2020 baseline survey, 28 (43%) reported a decreased intention to use EHR, 9 (14%) showed an increased intention, and another 27 (43%) maintained the same opinion.

**Figure 2.**
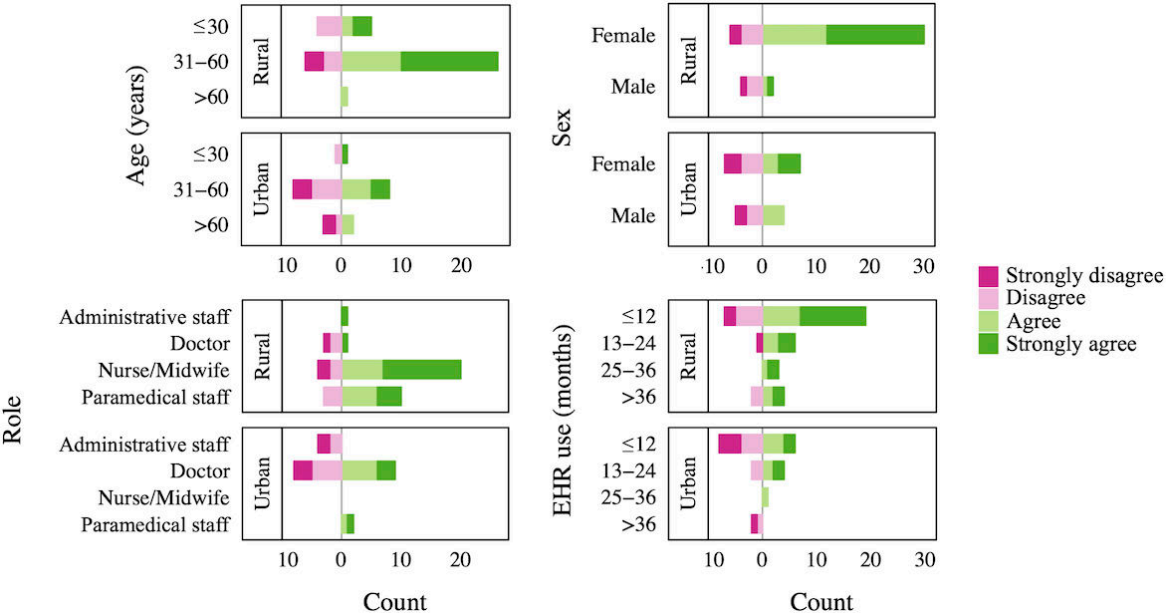
Total counts of responses to the question “I intend to use the EHR in the next 12 months” across different subgroups in 2022.

Subgroup analyses (Figure 2) of users from urban and rural sites, both of which transitioned to the Microsoft-based EHR by April 2021, revealed high levels of disagreement with continued EHR use among administrative staff and doctors in the urban setting. At the rural site, more doctors expressed disagreement compared to other roles. Urban site responses were symmetrically distributed with both positive and negative sentiments across age groups and sex. Conversely, in the rural setting, women and younger users expressed higher intent-to-use the EHR compared to men and older respondents. Acceptability was lowest among users with less than 12 months of EHR use.

### Content Analysis of User Feedback

The preliminary analysis of quantitative data raised the following questions for content analysis: (1) Why was acceptability different in the urban and rural sites despite using the same EHR system?; (2) Why was acceptability lower among doctors in urban and rural settings compared to other professions?; (3) Why was acceptability higher at the remote site despite similar, if not more, resource constraints compared to the rural site?; (4) Why was acceptability lower among less experienced users?

### Content Analysis of User Feedback

Common feedback included positive sentiments toward fundamental features such as record input (from administrative staff, pharmacists, and midwives), access to patient information (noted by midwives, nurses, and doctors), and data integration within the hospital system (mentioned by nutritionists and doctors). However, pervasive concerns about unresponsive interfaces were reported across all sites, attributed to slow laptop performance at the urban site and slow internet connectivity at rural and remote sites. Users also expressed frustration with system bugs and freezes, indicating a perceived lack of readiness for the Microsoft-based EHR implementation. Additionally, physicians in urban and rural sites, who used the Microsoft-based EHR, specifically commented on the interface complexity for having an excess of on-screen buttons (Table 2).

**Table 2.** Content analysis of unstructured responses from the acceptability surveys.

Categories	Data extracts
Useful core features, but repetitive and cumbersome interface	“Easy to find information of the patient” (rural, laboratory technician)“Slow opening of application and too many save buttons to click when closing the chart” (urban, physician)
Need for multidevice support and workflow integration	“Not cross-compatible with other devices (computer/mobile phone)” (rural, administrative staff)“I wish it was integrated with Appointlet or my email. I wish I could just click on a button to contact a patient through text or Viber or Zoom” (urban, physician)
Dependence on unreliable internet connectivity	“Slow when the internet is weak” (remote, midwife)
Lack of time and opportunity for system (re)training	“There was a change or an upgrade of the platform at one point and I just did not have the time to relearn” (urban, physician)

### Integrating Quantitative and Qualitative Findings

Disparities observed in acceptability were linked to both the demographic composition of survey respondents and the specific EHR system used. The urban setting had a higher concentration of physicians, while the rural site featured a mix of midwives and physicians, and the remote site had fewer physician respondents. Integrating quantitative and qualitative datasets yielded the following interpretations.

Despite using the same Microsoft-based EHR system, lower acceptability in the urban setting compared to the rural site can be attributed to several factors. Although urban respondents did not identify internet connection issues unlike their rural counterparts, demographic differences played a role. Urban site respondents were predominantly doctors, who displayed a more critical stance compared to other healthcare professionals.

Higher acceptability at the remote site, despite experiencing more resource constraints than the rural site, was influenced by the continued use of the OpenMRS-based EHR. Dissatisfaction at the rural site, particularly among doctors, was due to the lack of multidevice support of the Microsoft-based EHR. Common concerns across rural and remote sites included internet dependency and interface issues, although the remote site reported fewer problems with bugs and freezes with the OpenMRS-based EHR.

Investigating why acceptability was lower among physicians in both the urban and rural settings compared to other health care professionals revealed several insights. Flexibility was a success factor, with physicians emphasizing the need for a cross-platform EHR system that enabled easier access to patient information and integration with their workflow and other productivity tools. The Microsoft-based EHR’s restrictions led to additional workflow disruptions, including reliance on low-performance computers and intermediary note-taking methods (eg, pen and paper, personal computers)—an unintended consequence which arose from efforts to implement a desktop-based, privacy-focused design.

Beyond technical implementation barriers, social factors also influenced adoption. Exploring the lower acceptability among less experienced users highlighted the learning curve associated with new platforms and the need for periodic technical support. New users appreciated the transition from paper to electronic records but identified system navigation complexity as a concern, especially for those with limited technological literacy. Recommendations included a self-explanatory interface and a help window for direct support.

### Lessons Learned

Successful implementation of the EHR systems was facilitated by the active involvement of users, developers, and site managers. This collaboration allowed timely feedback cycles, leading to iterative improvements and the adoption of an integrated EHR used not only by physicians but also by nurses and midwives. The Microsoft-based EHR was intentionally designed to enable task-shifting: nurses would document and prescribe treatment plans for asynchronous physician review. In practice, this worked well in the rural site. However, the same approach proved less relevant in the urban site, where nurses typically worked alongside physicians. Acceptability declined in this setting after the EHR transition, as the initial system was locally installed. To address this, the Microsoft-based EHR was later migrated to a web-based platform that supported access from laptops and mobile devices.

The implementation also benefited from favorable policy timing. The COVID-19 pandemic in 2020 accelerated digital adoption out of necessity [8], while the Universal Healthcare Law (enacted in 2019) created legal incentives to adopt EHRs that complied with national reporting and claims processing standards [1]. These contextual enablers were not originally anticipated in the project’s planning phase, but enhanced uptake.

However, structural challenges persisted, particularly with internet connectivity. Although limited access was acknowledged during planning, the actual impact was more severe than expected. For example, the Microsoft-based EHR, which was reliant on stable broadband, could not be launched in to remote site. Internet service providers and EHR vendors did not have strong financial incentives to serve low-density areas. The PPCS experience showed that reframing paramedical staff as core users, not secondary ones, can strengthen the business case for scaling EHRs in underserved areas [7]. Nonetheless, government support remains essential to overcome the limited commercial viability.

Budget allocation was further complicated by the decentralized governance structure in the Philippines [23]. While some infrastructure costs were cofinanced by local government units, the actual continuity of support varied. One site discontinued EHR use following a change in local leadership, despite the original plan for sustainability beyond the pilot phase. With local elections occurring every three years, digital health programs remain susceptible to political turnover [24]. This introduces unpredictability that can deter significant upfront investments in infrastructure and innovation.

## Discussion

This study described the implementation strategy, intervention components, and the evolving acceptability of two EHR systems over a three-year period across three primary care settings. Acceptability declined notably in both urban and rural sites following the transition to a Microsoft-based EHR. Conversely, the remote site, which continued using a OpenMRS-based EHR, consistently reported high levels of acceptability. User feedback highlighted doctors’ preference for a more flexible EHR that could operate across various devices. Paramedical staff pointed out recurrent bugs in the Microsoft-based EHR, suggesting premature implementation. The transition between EHR systems posed challenges, necessitating users to adapt to new interfaces, with inadequate support identified as a significant barrier.

The study’s outcome measurement approach had several limitations. First, the absence of in-depth interviews and direct observation limited the ability to capture richer insights, particularly from users who may have opted out of system use. Second, is the low response rate from 2020 to 2022, likely influenced by the online format of the survey. This may have introduced selection bias and skewed the results toward participants with higher technological capacity.[25] While the rates in our study were higher than those from similar surveys [26-28], the views of nonrespondents could differ meaningfully [26-28]. Additionally, the COVID-19 pandemic in 2020 drastically altered the health care landscape and may have influenced respondents’ sentiments towards the EHR systems.

Efforts to scale up EHRs in primary care should recognize the complex interplay between system design, user needs, and contextual factors. A user-centered model designed for a rural context may not seamlessly apply to an urban setting. Consequently, adopting a single system nationwide risks replicating the misalignments observed in this program. Future national strategies should balance standardization with flexibility, allowing for context-sensitive adaptations to maximize EHR use across varied health system settings.

## Supplementary material

10.2196/63036 Checklist 1 iCHECK-DH (Checklist for the Reporting on Digital Health Implementations) guidelines.

10.2196/63036 Checklist 2 Good Reporting of A Mixed Methods Study reporting standards.

## Abbreviations

EHR: electronic health record
OpenMRS: Open Medical Record System
PPCS: Philippine Primary Care Studies
