# Supplementary material for: Implementing Electronic Health Records in Philippine Primary Care Settings: Mixed-Methods Pilot Study
Source: JMIR Med Inform. 2025 Jul 15;13:e63036. doi: 10.2196/63036 (PMC12283060; doi:10.2196/63036)
Supplement: Checklist 1 [file medinform-v13-e63036-s001.docx]

Appendix 1. iCHECK-DH Checklist

| **Section** |  | **Item** | **Description** | **Section, Page** |
| --- | --- | --- | --- | --- |
| **TITLE** | 1 | Title (M) | Identify as an implementation report and describe the implementation in the title, keywords, or both. | Keywords, p. 2 |
| **ABSTRACT** | 2 | Abstract (M) | Provide a summary of the key elements of the implementation report, including a description of the implementation strategy and the intervention, defining the key elements of the implementation and health outcomes, and specify the key performance indicators (KPIs)/outputs. | Abstract, pp. 1-2 |
| **INTRO-**  **DUCTION** | 3 | Context (M) | Describe the geographical areas, organizations, target populations, and implementation context. Consider social, cultural, economic, political, health care, and organizational barriers; infrastructures; and facilitators that may influence implementation elsewhere. Explicitly highlight whether a national digital health strategy exists and whether implementation is aligned with the strategy.  Describe the stage of the implementation (developing or adapting solution/piloting and evidence generation/package and advocacy/acceleration/deploying/scaling up/hand over or complete). | Introduction, p. 2 |
|  | 4 | Problem Statement (M) | Describe the health care or public health problem, challenge, or deficiency that the implementation aims to address. (If applicable, include a reference to the “health system challenge” of the WHO Classification of Digital Health Interventions in the description.) | Introduction, pp. 2-3 |
|  | 5 | Similar Interventions (M) | Mention whether this implementation was inspired by another existing one. If so, what is the added value of your intervention, if any, compared to the initial one, and what, if anything, has been done differently? | Introduction, p. 3 |
| **METHODS** | 6 | Aim and Objectives (M) | Describe the main objectives and the overall aim of the implementation. Describe how these will be measured using predefined primary and secondary outcome(s) and KPIs for this implementation and the expected intervention(s). | Methods, p.3 |
|  | 7 | Blueprint Summary (M) | Describe the design and key features of the intervention and key points of the implementation strategy and roadmap. | Methods, p. 3 |
|  | 8 | Technical Design (M) | Specify reasons for developing or choosing this tool. Does it combine several tools? Provide a brief description of the tools (functionality and architecture) and how it fits into the health enterprise architecture and investment roadmap (if applicable). Indicate whether the solution is based on an existing solution or has been developed or purchased specifically for this intervention.  Describe the type of technology used (eg, artificial intelligence [AI] applications) and the license of the technology (open source, free, commercial, intellectual property [IP] ownership, etc) and include code documentation (if available), a link to the application, and a link to wiki or the project website. | Methods, pp. 3-4 |
|  | 9 | Target (M) | The target refers to the focus or recipient of the intervention. It is the individual, group, system, or problem that the intervention aims to change or improve. Describe the characteristics of the targeted “site(s)” (locations, staff, resources, etc) for implementation and any eligibility criteria, as well as the population targeted by the intervention and any eligibility criteria. | Methods, p. 4 |
|  | 10 | Data (M) | Describe the data governance, including the life cycle (collection, processing, storage, modification, sharing, suppression); data ownership (mention whether patients actually have access to the data); data protection measures; confidential use of routine data; expected level of data integration; data for research; cross-border data agreement, if any; the applicable legal framework; and how the project complies with it. Describe data consent: Has patient consent been obtained? Describe the approach to data protection and cybersecurity (eg, security by design, privacy by design) and where the data are hosted (eg, in-country, cloud-based, hybrid model). If applicable, describe the government’s data policy. | Methods, p. 4 |
|  | 11 | Interoperability (M) | Describe the interfaces (what other systems does the tool connect to) and the standards that were used (which specific ones and rationale of choice; eg, semantic ontologies, such as the International Classification of Diseases [ICD], Systemized Nomenclature of Medicine – Clinical Terms [SNOMED CT], Logical Observation Identifiers, Names and Codes [LOINC], or technical standards, such as Health Level Seven Fast Healthcare Interoperability Resources [HL7 FHIR]). | Methods, p. 4 |
|  | 12 | Participating Entities (M) | Describe the following:  Implementing organization(s): type of organization(s), mission, leadership, vision, etc.  Government involvement: Describe whether the government was involved in the implementation, at what level, and at what stage(s).  Partners: Describe all partners (organizations) and their role in the implementation.  Funders: List all actors and stakeholders who have funded or invested in the development of the implementation (if different from the implementation, eg, using an existing digital health intervention). Indicate their level of involvement in terms of funding.  Mention which entity will own the final product and intellectual property after the implementation phase. | Methods, pp. 4-5 |
|  | 13 | Budget Planning (M) | Describe the planned budget for implementation (include costs such as change management, user training, project management, technology pricing, total cost of ownership). If possible, include actual costs, otherwise describe the range or percentage of the total budget. Indicate the time frame covered by the budget. Describe the budget for the intervention (eg, development, purchase or adaptation of a free tool); if possible, include actual costs, otherwise describe them as a percentage of the total budget. | Methods, p. 5 |
|  | 14 | Sustainability (M) | Describe the business model, including the sustainability model (financial, environmental, etc). If possible, relate outcomes to costs to assess sustainability. Describe long-term exit strategies and all dimensions considered to sustain the project after the end of the funding period. If applicable, describe the potential institutionalization of the project. | Methods, p. 5 |
| **RESULTS** | 15 | Coverage (M) | Describe whether the coverage of implementation is international, national, regional, or at the level of, for example, municipalities. If coverage is subnational, describe the regions. Provide information about the relative importance of the coverage (eg, percentage of the eligible population covered). | Results, pp. 5-6 |
|  | 16 | Outcomes (M) | Describe the primary and other outcomes of the implementation. Detail the actual outcomes, using the predefined outcome measures (if applicable). | Results, pp. 7-10 |
|  | 17 | Lessons Learned (M) | Describe any lessons learned from the implementation experience that could be used to improve future outcomes. This could include, but is not limited to, success factors, implementation challenges, or budget considerations.  Success factors: Describe factors that positively influenced the implementation (eg, involvement of key stakeholders). In addition, describe contextual factors that may have positively influenced the results (eg, new legislation that facilitated adoption).  Challenges to implementation: Describe challenges (process related, such as resistance to change, but also technical). Include contextual factors that may have affected the achievement of outcomes, such as an unexpected change of government or “opposing key players” who, despite potential participation, may hinder implementation (eg, software companies managing regional digital health may act as barriers to innovation).  Budget: Describe whether the implementation budget was met, and if not, why not. In addition, detail the expected operational costs (eg, licensing, maintenance, human resources, updates to in-house developments) to estimate the total cost of ownership. Include actual costs, otherwise describe them as a percentage of the total budget.  What recommendations can be drawn from the lessons learned? | Results, pp. 10-11 |
|  | 18 | Unintended Consequences (NM) | Describe any unintended consequences (positive or negative), harms, or negative side effects (if any). | Results, p. 10 |
| **DISCUSSION** | 19 | Discussion (M) | Provide a summary of the conclusions and future implications. | Ethical Considerations, p. 12; Conflicts of Interest, p. 12 |
| **GENERAL** | 20 | General (NM) | If applicable, include statements on regulatory approvals (eg, as appropriate, ethical approval, governance approval), trial or study registration (availability of protocol), and conflicts of interest. For implementation reports with a research component, ethical approval or a waiver from an appropriate ethics committee is required. For those without a research component, ethical considerations may still be relevant but do not necessarily require approval or a waiver. Authors may consult Eccles et al for further guidance on ethical considerations in their specific context. | Acknowledgements, p. 12; |
