# Supplementary material for: Implementing Electronic Health Records in Philippine Primary Care Settings: Mixed-Methods Pilot Study
Source: JMIR Med Inform. 2025 Jul 15;13:e63036. doi: 10.2196/63036 (PMC12283060; doi:10.2196/63036)
Supplement: Checklist 2 [file medinform-v13-e63036-s002.docx]

Appendix 2. GRAMMS Reporting Guidance

| **Guideline** | **Section, Page** |
| --- | --- |
| Describe the justification for using a mixed methods approach to the research question | Methods, p. 5 |
| Describe the design in terms of the purpose, priority and sequence of methods | Methods, p. 5 |
| Describe each method in terms of sampling, data collection and analysis | Methods, p. 5 |
| Describe where integration has occurred, how it has occurred and who has participated in it | Methods, p. 5; Results, p. 10 |
| Describe any limitation of one method associated with the present of the other method | Methods, p. 5 |
| Describe any insights gained from mixing or integrating methods | Methods, p. 5; Results, p. 10 |
